# Immune correlates underlying small fiber neuropathy presenting as vaccine-associated post-acute SARS- coronavirus syndrome

**DOI:** 10.3389/fimmu.2026.1752120

**Published:** 2026-03-19

**Authors:** Alessandro Limongelli, Federica Bozzano, Alireza Hajabbas Farshchi, Margherita Bellucci, Martina Bavastro, Chiara Castellano, Giampaola Pesce, Francesca Antonini, Alex Incensi, Maria Pia Giannoccaro, Antonio Uccelli, Genny Del Zotto, Lorenzo Moretta, Vincenzo Donadio, Luana Benedetti, Andrea De Maria

**Affiliations:** 1 Università degli Studi di Genova, Department of Health Sciences, Genoa, Italy; 2 Istituti di Ricovero e Cura a Carattere Scientifico (IRCCS), Ospedale Policlinico San Martino, Genova, Italy; 3 Department of Neuroscience, Rehabilitation, Ophthalmology, Genetics, Maternal and Child Health, University of Genova, Genoa, Italy; 4 Integrated Department of Services and Laboratories, IRCCS Istituto Giannina Gaslini, Genova, Italy; 5 IRCCS Istituto delle Scienze Neurologiche di Bologna, UOC Clinica Neurologica, Bologna, Italy; 6 Tumor Immunology Unit, Bambino Gesù Children’s Hospital, IRCCS, Rome, Italy; 7 S.C. Malattie Infettive, Azienda Sociosanitaria Ligure n.5, La Spezia, Italy

**Keywords:** innate-like T cells, Long-COVID, NKG2D, PASC, post-acute sequelae of SARS-CoV-2 infection, SARS-CoV-2, small fiber neuropathy, vaccine

## Abstract

**Background:**

A spectrum of adverse events overlapping with Post-acute Sequelae of SARS-CoV-2 infection (PASC) occurs in some patients following SARS-CoV-2 vaccination including small fiber neuropathy (SFN) and cognitive symptoms.

**Aims:**

Accruing information regarding disease course and immune response imbalances in these patients.

**Methods:**

We studied 71 previously healthy patients with neurological symptoms following SARS-CoV-vaccination. All had negative neurological workup for central/peripheral involvement (MR, EMG/EN). Cutaneous biopsy (21pts.) and peripheral blood sampling (20pts) were performed for anti-idiotype Ab analysis (ACE-2,NRP-1) (ELISA, IF) and for Flowcytometric analysis.

**Results:**

Paresthesia, cognitive impairment and autonomic symptoms agreed with SFN international definition. Comparative differences included abrupt onset, presence of simultaneous diverse paresthesia across multiple body regions frequently affecting the facial and cervical regions (44%) and the trunk (26%), associated to dysautonomia. Median time from vaccination to symptom manifestation was 3 days (mean ± SD: 8.76 ± 17.4 days). Symptom severity was still high (5.9 ± 1.9 mean+SD) at the time of evaluation and sampling, (382 ± 133 days from onset. Reduced small fiber density was observed in 19/21 biopsies. Anti-ACE-2 antibodies in 9/71pts. (12%) and 4/19 (21%) vaccinated HD sera and NRP-1 reactivity in 14/71 (20%) patient and 1/19 (5%) HD sera were not significantly increased. Peripheral NKG2D+CD8+ and NKG2D+DNAM-1+CD4+ T-cells were increased. Circulating inflammatory CD34+ cells were increased and generated *in vitro* a prevalence of NKG2D+DNAM-1+ T-cells.

**Conclusion:**

PASC-vac SFN is associated with persistent immune imbalances common to other immune-mediated diseases. Additional effort to identify immune mechanisms unleashing PASC-vac SFN will contribute to modulate future early interventions for these patients and refine vaccine design.

## Introduction

Symptomatic or pauci-symptomatic SARS-CoV-2 infection can be followed by a clinical syndrome defined as Long-COVID or Post-Acute Sequelae of SARS-CoV-2 infection (PASC) (1, 2). This condition is characterized by the persistence or *de novo* appearance of symptoms after SARS-CoV-2 infection, which may involve the central and peripheral nervous systems, the cardiovascular system (including pericarditis, myocarditis, and coagulopathies), dysautonomic symptoms of the gastrointestinal or cardiovascular systems, myalgias, and fatigue (3–5). Its incidence in the general population is estimated at 7% in the US as estimated by a National Health Interview Survey in 2022 (2) and it has been found to affect 35-57% of patients with symptomatic COVID-19 (6, 7).

A spectrum of adverse events substantially overlapping with PASC has been reported in a minority of patients following SARS-CoV-2 vaccination (PASC-vac). These include central and peripheral nervous system syndromes such as paresthesia, small fiber neuropathy, Guillain-Barré syndrome, and cognitive symptoms (including brain fog, attention, and concentration deficits) (8–11), as well as pericarditis, myocarditis, dysautonomic gastrointestinal, genitourinary, and cardiovascular symptoms, all accompanied by persistent fatigue and myalgias (12–14) While cardiovascular symptoms of PASC-vac occur with a frequency at least 1–2 orders of magnitude lower compared to those observed after wild-type infection and have been well characterized for the cardiovascular system (14–17), the incidence of small fiber neuropathy (SFN) and brain fog -acknowledged in Long-COVID or PASC - is poorly recognized or neglected in PASC-vac, as these were not listed or actively sought as potential adverse events of SARS-CoV-2 vaccines. The mechanisms leading to Post-Acute Sequelae of SARS-CoV-2 infection (PASC), whether virus- or vaccine-induced, represent an active focus of scientific interest. Both SARS-CoV-2 and current SARS-CoV-2 vaccines share a spike receptor-binding domain (RBD), that has been shown to bind to the two viral receptors, ACE-2 (18) and NRP-1 (19) (20). ACE-2 and NRP-1 are widely distributed in multiple organ/systems including vascular, nervous, and immune cells (21).

Neurological involvement during COVID-19 has been proposed to occur either through direct neurovascular infection or be mediated by SARS-CoV-2-derived structural proteins, leading to neuroinflammation or direct infection of neurological structures (22–26). Prolonged virus persistence after wildtype infection at autopsy and in some patients (26, 27) supports the hypothesis that PASC may be sustained by the release of structural proteins or by persistent antigenic stimulation of the immune system, causing inflammatory effects on the nervous and cardiovascular systems.

The mechanisms underlying post-vaccine PASC (PASC-vac) have, to date, received limited attention. They have been proposed to be similarly associated with either persistent immune activation leading to immune-mediated symptoms or the generation of anti-idiotype antibodies (28). Indeed, for PASC-vac, the persistence of the SARS-CoV-2 spike protein produced by mRNA vaccines in some patients raises the possibility of sustained or prolonged immune stimulation, resulting in immune-mediated systemic symptoms (27, 29–31).

Given the substantial lack of information regarding potential specific or general immune response imbalances in patients with PASC-vac and neurological clinical manifestations, we studied a cohort of patients. We employed clinical examination, cutaneous biopsy, anti-idiotype antibody assessment, and peripheral blood immunophenotyping to gather data on the overall clinical characteristics of SFN, its timing, and immune features in patients affected by PASC-vac syndrome. Our results provide an overview of the spectrum and prevalence of symptoms related to peripheral nervous system involvement in affected patients, offer insights into the relatively low frequency of anti-idiotype antibodies, and indicate that a significant skewing of peripheral blood T-cell populations and increased CD34+ inflammatory precursor cell circulation is associated with this condition, even months to years after symptom onset.

## Materials and methods

### Patients

Patients presenting with peripheral neurological symptoms, including possible peripheral neuropathy, after either wild-type SARS-CoV-2 infection or SARS-CoV-2 vaccination (using recombinant vaccines) were seen at our institution’s Infections in Immunocompromised Host Unit or Neurology Unit. The study was approved by the Regional Ethical Committee (n°CET-Liguria 555/2023-DBid13545). Sequential patients presenting with PASC Syndrome either post-viral or post-vaccine that included paresthetic and/or dysautonomic symptoms were considered. Informed consent was obtained from all individual participants included in the study. The present evaluation includes only those experiencing symptom onset after vaccine administration which began between February 2021 and September 2022. Patients with prior COVID-19 or neurological abnormalities detected during the visit, or those with findings on cerebral or spinal MRI, EEG, or EMG attributable to CNS lesions or radiculopathies/mono- or polyneuropathies, were excluded from further evaluation. Vaccines included: Pfizer Comirnaty (n°43), Moderna (n.15), AstraZeneca (n.5), J&J (n.2) Heterologous Mix (n.6).

For each patient, a detailed history was taken, including the timing of vaccination, symptom appearance, intensity of discomfort, and impact on leisure, sports, and work activities. Twenty-five milliliters (25 mL) of peripheral blood was drawn during the first visit. Participants were counselled skin biopsy for diagnostic purposes, but were permitted to opt out of the procedure for socio-economic, clinical, or logistical reasons; consequently, the biopsy rate in our cohort reflects the real-world availability and patient acceptance of skin biopsies in routine clinical practice in our area. All patients provided informed consent.

### Immunofluorescence

Immunofluorescence screening for anti-NRP1 autoantibodies was performed using a commercial tissue-based assay (Euroimmun, Germany). Briefly, monkey cerebellum slides were incubated with patient serum diluted 1:10, followed by fluorescein isothiocyanate (FITC)-conjugated goat anti-human secondary antibodies (Euroimmun, Germany). A positive control was included using anti-NRP1 monoclonal antibodies at 2 µg/mL (Santa Cruz Biotechnology). Fluorescence was visualized and recorded with a manual microscope (EurostarIII Plus, Euroimmun, Germany).

### Immunofluorescence confocal microscopy

Fluorescence confocal imaging was performed using a Leica Stellaris 8 Falcon TauSTED inverted confocal microscope (Leica Microsystems, Mannheim, Germany). Excitation was provided by an 80 MHz supercontinuum white light laser, with wavelengths of 488 nm and 638 nm selected via an acoustic-optical tunable filter (AOTF).

Fluorescence detection utilized two HyD S power detectors in analog mode. Detection ranges were set to 498–598 nm for 488 nm excitation and 650–720 nm for 638 nm excitation, respectively. Samples were imaged using a plan-apochromatic oil immersion 20x/0.70 NA objective at a speed of 600 Hz, with the pinhole set to 1.0 Airy unit. Images were acquired at 1024x1024 pixels. The Leica LAS X application Suite software package (version 4.4.0.24861) was used for image acquisition, storage, and visualization.

### Skin biopsy

Skin biopsies were performed on calf and thigh areas without signs of ongoing inflammation. Fifty μm sections from the same skin sample were obtained during the cryostat session (CM 1950; Leica, Deerfield, IL). Twelve free-floating sections were incubated overnight with a panel of primary antibodies, including the rabbit pan-neuronal marker protein gene product 9.5 (1:500; Abcam, Cambridge, UK, cat. no. ab108986) and mouse collagen IV (ColIV, 1:800, Chemicon, Temecula, CA, USA, cat. no. MAB1910). Sections were then washed and secondary antibodies, labeled with mouse Alexa Fluor(R) 488 (1:400; Jackson ImmunoResearch, West Grove, PA, USA; n. 715-545-150) and rabbit cyanine dye fluorophores 3.18 (1:800; Jackson ImmunoResearch, West Grove, PA, USA; n. 711-165-152) were added for an overnight incubation. Epidermal nerve fiber density (ENFs: number of unmyelinated fibers per linear millimeter of epidermis) was calculated by considering a single epidermal nerve fiber marked by PGP 9.5 crossings of the dermal–epidermal junction. To study the innervation pattern digital images were acquired by a laser-scanning confocal microscope (Leika DMIRE 2, TCS SL, Leika Microsystems, Heidelberg, Germany). Each image was collected in successive frames of 1-2 μm increments on a Z-stack plan at the appropriate wavelengths for secondary antibodies with a x20 or x40 plan apochromat objective and subsequently projected to obtain a double-stained digital image by a computerized system (LCS lite, Leica Microsystems, Heidelberg, Germany). The diagnostic yield of skin biopsy in evaluating somatic innervation abnormalities was estimated by the area under the receiver operating characteristic (ROC) curve. For all analyses, significance was assumed for p < 0.05.

### Serum anti-ACE2 evaluation

The presence of anti-ACE2 IgG the serum has been evaluated by ELISA assay (Eagle Bioscience, Amherst, USA) according to manufacturer instructions.

### Flow cytometry

**Peripheral Blood Mononuclear Cells (PBMCs)** were isolated by Ficoll–Paque gradient centrifugation and cryopreserved at –80 °C until further processing. Cells were analyzed by multicolor flow cytometry using direct staining with fluorochrome-conjugated mouse anti-human monoclonal antibodies (mAbs). PBMCs were gated based on forward and side scatter parameters (FACS Fortessa, BD, Mountain View, CA, USA; 10,000 events collected) as previously described (32).

**Antibodies:** A complete list of all purchased mAbs is provided in **Supplementary Table 1**. All mAbs were used at a final concentration of 1 µg/mL.

**Statistical Analysis:** Statistical analysis was performed using GraphPad Prism 9 and JMP 9 software. Group comparisons were performed using the two-tailed Fisher’s exact test, and nonparametric data were analyzed using the Mann-Whitney U-test. A p-value of 0.05 was set as the threshold for statistical significance.

## Results

### Phenotypic characterization of post-acute sequelae of SARS-CoV-2 vaccination syndrome in a sequential patient cohort

Our investigation initiated with a precise characterization of the clinical manifestations in a sequential cohort of patients diagnosed with Post-Acute Sequelae of SARS-CoV-2 Vaccination (PASC-vac) syndrome. Rigorous inclusion criteria mandated that all subjects were immunocompetent and clinically asymptomatic prior to the administration of their initial vaccine dose, with no documented history of oncological, vascular, or established autoimmune pathologies. Furthermore, their pre-vaccination status indicated unhindered engagement in professional and recreational activities. A crucial exclusionary criterion was the absence of any discernible structural or functional abnormalities as evidenced by MRI, CT, and comprehensive electrophysiological assessments.

The analyzed cohort comprised 71 subjects (47 females, mean age ± SD 45.5 ± 11.22 years; 24 males, mean age ± SD 45.6 ± 18.11 years). A salient clinical feature observed was profound fatigue, frequently co-occurring with myalgia. While reported with a higher, albeit non-significant, frequency in female patients, this debilitating symptom consistently correlated with a marked impairment in ambulation, performance of activities of daily living (ADLs), professional duties, and engagement in physical exercise. Illustratively, a previously highly conditioned military cadet experienced functional limitations precluding service continuation, and eight healthcare professionals (nurses, psychologists, physiotherapists) necessitated prolonged medical leave.

Analysis of autonomic manifestations, including paroxysmal tachycardia unrelated to exertion or nocturnal tachycardia accompanied by dizziness, revealed that despite initial numerical discrepancies, the relative incidence rates demonstrated no significant gender-related variance upon adjustment for subgroup size (**Table 1**). Correspondingly, other systemic symptoms, such as cognitive dysfunction (‘brain fog’), orthostatic hypotension with concomitant dizziness, gastrointestinal dysfunctions, tinnitus, and various respiratory, vasomotor, or cutaneous disturbances, also exhibited comparable frequencies across genders (**Table 1**).

**Table 1:** Findings and symptoms of 71 patients with PASC (post-vaccination).

Symptoms	Number (%)	Age Range (mid)	F. N°(%)	M. N° (%)	P value
Dysautonomic	61(87%)	25-71(48)	43 (71)	18 (30)	0.0065(**)
Orthostatic	25(35%)	25-68(46)	18 (72)	7 (15)	ns
Vasomotor	23(88%)	27-69(48)	18 (78)	5 (22)	ns
Brain fog	29(41%)	25-79(52)	19(66)	10 (34)	ns
Fatigue	22(30%)	25-79(52)	15 (68)	7 (32)	ns
Tinnitus	20(28%)	25-68(46.5)	11 (55)	9 (45)	ns
Dyspnea	14(20%)	27-71(49)	8 (57)	6 (42)	ns
Gastrointestinal	14(20%)	28-58(43)	6 (43)	8 (57)	ns
Nausea	6 (8%)	26-38(32)	4 (67)	2 (33)	ns
Diarrhea	6(8%)	27-71(49)	2 (50)	2 (50)	ns
Irregular bowel	6 (8%)	28-58(43)	2 (50)	2 (50)	ns
Constipation	4 (7%)	25-52(38.5)	2(50)	2 (50)	ns
Skin manifestation	12 (17%)	25-59(42)	10 (83)	2 (17)	ns
Urinary symptoms	2 (3%)	26-37(31.5)	0(0)	2 (100)	ns

F, female; M, male.**p<0.01.

Subsequent to symptom genesis, an abrupt emergence of paresthesias was uniformly observed across the patient cohort, exhibiting progressive dissemination to diverse anatomical regions. These sensory disturbances were qualitatively described as deep pain, burning dysesthesia, or tingling sensations, primarily localized to the extremities (limbs, hands, and feet), with no discernible gender-related predilection (**Table 2**). Of particular diagnostic significance was the atypical topographical distribution of these paresthesias, frequently affecting the facial and cervical regions (44%) and the trunk (26%), which deviates markedly from the classical ‘glove-and-stocking’ pattern typically observed in established mono- or polyneuropathies or conventional small fiber neuropathy. Further evidence of peripheral nervous system involvement included thermal dysesthesias (e.g., aberrant thermosensation during bathing or pain induced by environmental temperature shifts) and hyperesthesia of the digital extremities (e.g., heightened tactile sensitivity to common textures like paper), present in 45% of patients and statistically more prevalent in females (p=0.0228, Fisher’s exact test, **Table 2**).

**Table 2:** Peripheral neuropathic symptoms in 71 patients with PASC (post-vaccination).

Symptoms	Total Number (%)	Age (mid)	Females Number (%)	Males Number (%)	P value
Lower Limb paresthesia	56(78.87%)	26-79(52.5)	38(67.86%)	18(32.14%)	ns
Upper Limb paresthesia	41(57.74%)	28-68(48)	33(80.49%)	8(19.51%)	0.0048(**)
Facial and cervical paresthesia	31(43.66%)	27-68(61)	23(74.20%)	8(25.80%)	ns
Alteration in tactile sensitivity	32(45.07%)	25-82(53.5)	26(81.25%)	6(18.75%)	0.0228(*)
Thoracic paresthesias	18(25.80%)	27-58(42.5)	13(72.22%)	5(27.77%)	ns

*p<0.05; **p<0.01.

Notably, these paresthesias often did not occur in isolation. Patients frequently reported a concurrent presentation of diverse paresthetic sensations simultaneously across multiple body regions. As illustrated in **Table 2**, common manifestations included both upper and lower limb involvement, along with significant distribution in the face/neck and trunk areas.

### Temporal symptom intensity and symptom distribution and correlation with skin biopsy findings

Of the 71 symptomatic patients enrolled in this study, 64 experienced the first manifestations of their disease following the initial vaccine dose. Overall, 22 patients discontinued their vaccination schedule due to the onset of PASC-vac symptoms after the first dose. Among the remaining participants, 30 received two vaccine doses, and 19 s completed the vaccination schedule despite the appearance and persistence of their symptoms.

**Figure 1:**
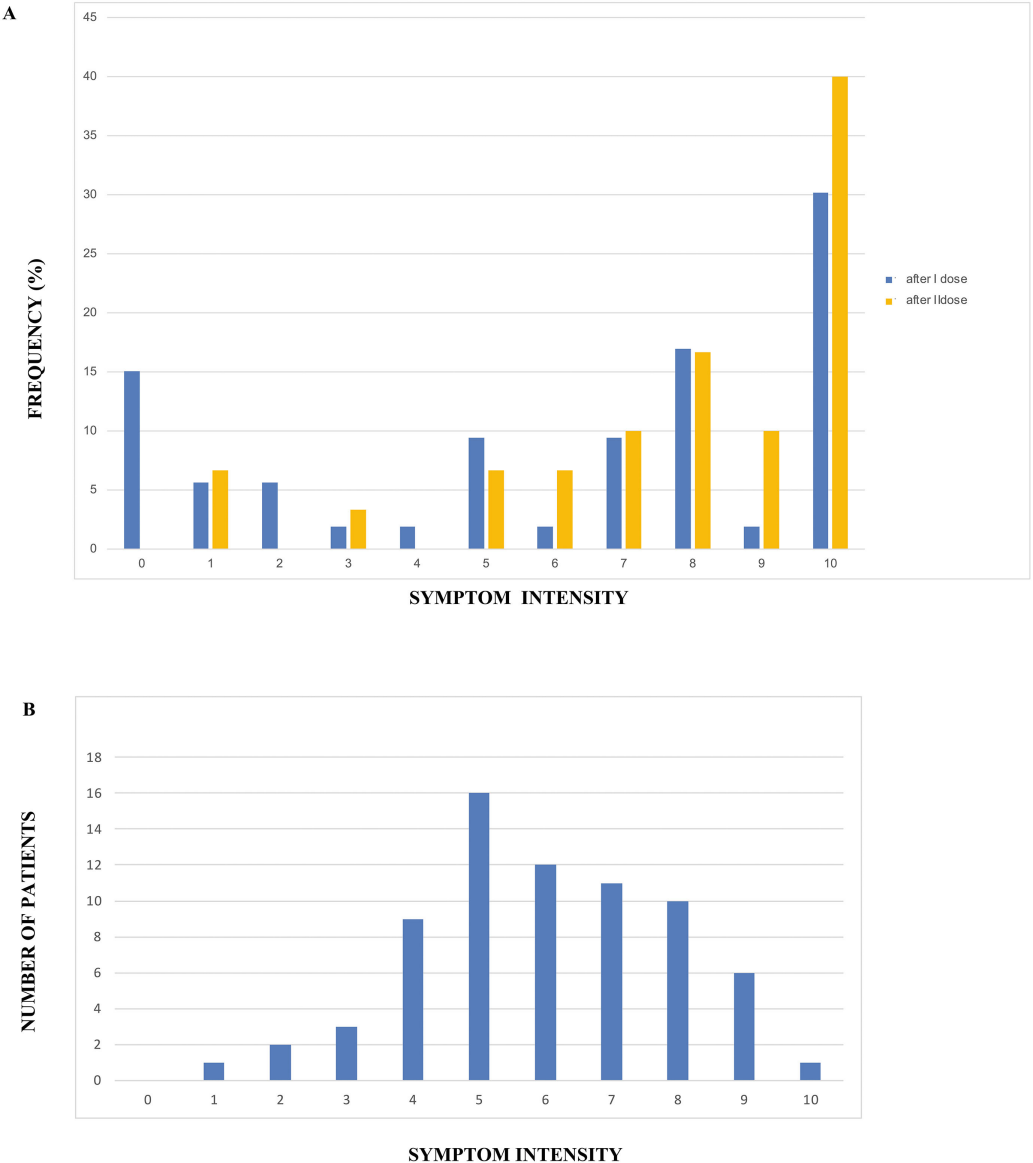
Frequency and symptom intensity scores within the cohort. Intensity scores according to a patient-defined 10-point Likert scale. Horizontal: Likert scale; Vertical: n° of patients with given symptom intensity. **(A)** Frequency and Symptom intensity score after 1^st^ and 2^nd^ vaccination (1-2-3) in patients with PASC-vac. **(B)** Symptom intensity score at the time of first clinical evaluation of 71 patients.

Regarding the temporal onset of symptoms post-vaccination, the median time to symptom manifestation was 3 days (mean ± SD: 8.76 ± 17.4 days). In patients who received a second dose (69% of the cohort), an immediate exacerbation of symptoms was frequently observed, often occurring on the same day (**Figure 1A**).

To quantify symptom severity, patients were asked to provide a 10-point Likert scale rating (10 being the most severe, 0 being asymptomatic) for symptoms experienced after each vaccine dose. At the time of clinical evaluation, patients also provided a similar rating for the persistence of symptom severity.

The mean time from symptom onset to actual clinical evaluation was 382 ± 133 days, indicating persistence of symptoms well beyond 3 month from onset. At the time of evaluation, reported symptom severity ranged from 1 to 10, with a normal distribution and a mean of 5.9 ± 1.9 (**Figure 1B**).

Analysis of symptom onset and severity revealed that 59% of patients reported severe symptoms (Likert scores 8-10), with 27% reporting maximal severity. The mean pain/symptom score after the first dose was 7.42 ± 2.74. In patients who discontinued vaccination after a single dose (n=22), PASC-vac symptoms were the primary cause of cessation. Among those who received a second dose, symptom severity remained high, with 65% reporting scores of 8-10.

**Figure 2:**
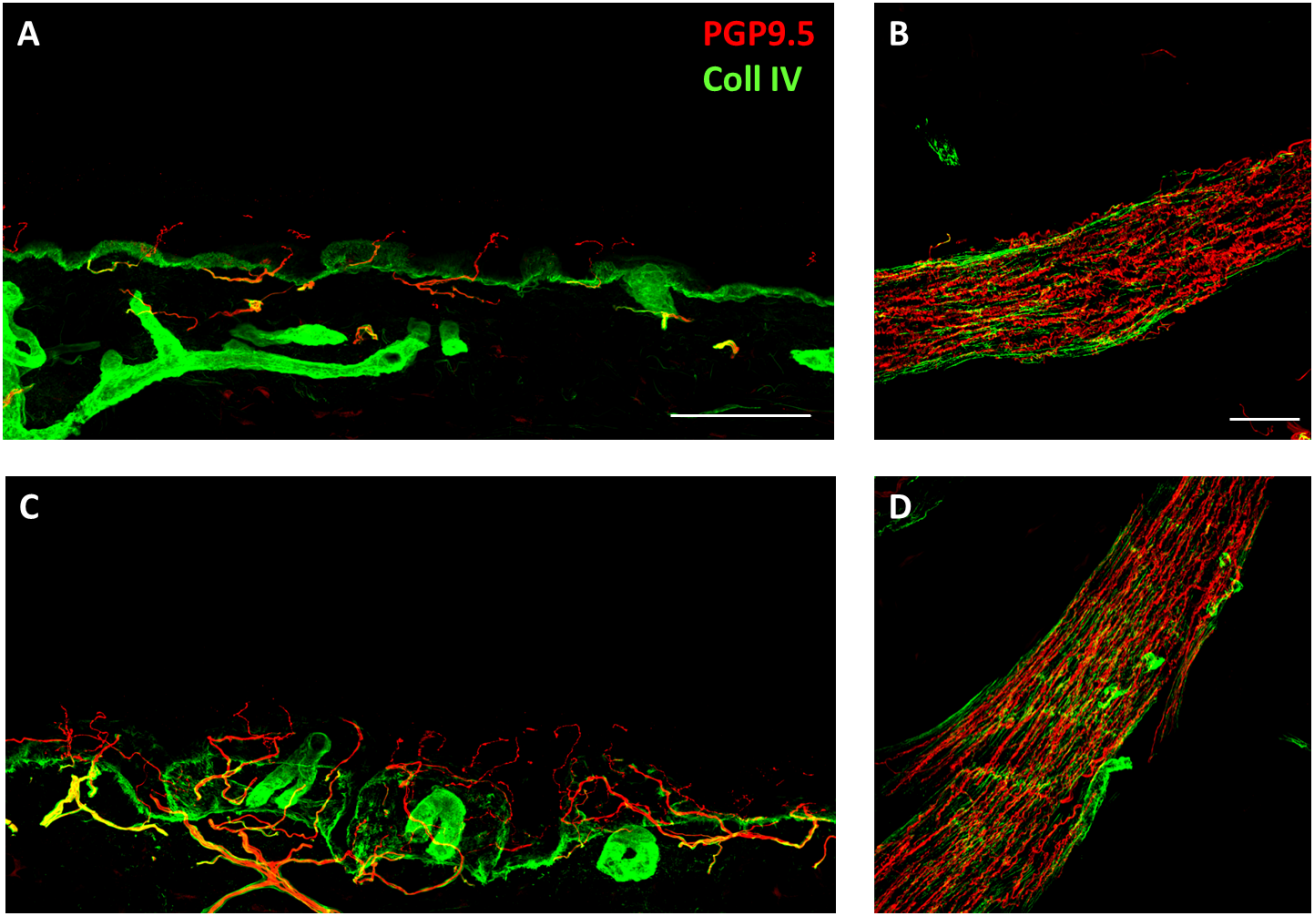
Somatic and autonomic patterns of innervation in a representative healthy control subject and a representative patient with small fiber neuropathy after vaccination against Covid-19. Epidermal and autonomic innervation disclosed by confocal microscope (x20) in the patient complaining neuropathic pain after vaccination against Covid-19 **(A, B)** and an age-matched healthy control **(C, D)**. Nerve fibers are marked in red whereas the collagen staining is shown in green. **(A-C)** Free-ending PGP9.5 immunoreactive nociceptive fibers are evident in the epidermis but they show a decreased density in the patient **(A)** compared to the healthy control **(C)**. This finding underlies a somatic small fiber neuropathy in this patient. The basement membrane separating epidermis from dermis is marked by collagen staining. **(B-D)** Muscle arrector pilorum shows a rich density of fibers running in a longitudinal and wavy pattern in the control subject **(D)**. The patient **(B)** shows a similar density autonomic fibers running in the muscle arrector pilorum although their pattern of innervation is less regular than that of the control with several fibers presenting irregularities and tortuosities along their course. This picture identifies subtle autonomic alterations.

Given the symptom profile consistent with Small Fiber Neuropathy (SFN), patients were referred for skin biopsy to confirm the diagnosis. However, access to skin biopsy was limited to a subset of cases for socio-economic, clinical, or logistical reasons. Skin biopsies were performed in 21 patients, revealing evidence of SFN in 19 cases, characterized by reduced small fiber density relative to age- and sex-matched norms (**Figure 2**).

The assessment of both proximal and distal cutaneous sites is critical to determine whether the pattern of nerve involvement is length-dependent (predominant distal involvement) or non-length-dependent (primary proximal involvement). A non-length-dependent pattern is typically observed in neuropathies of autoimmune etiology (33, 34). In our cohort, this pattern was found in 33% of patients, supporting an autoimmune origin in a subset of the population. This finding aligns with the “central” and “face-scalp” distribution of paresthesia reported respectively by 25 and 44% of patients in this study.

In this regard, it should be noted that biopsies were performed as standard procedure on the calf and thigh, irrespective of the distribution of symptoms. Thus, patients with “central” or face-scalp” paresthesia were found to have a “central” SFN based on thigh and calf biopsies.

To mitigate potential selection bias, and account for confounding variables between those who underwent biopsy and those who did not, we conducted a comparative analysis of baseline clinical characteristics and reported symptoms. No significant differences in symptom spectrum were observed between the two groups (**Supplementary Tables 1** and **2**). This suggests that the non-biopsied population presented with a clinical phenotype similar to the biopsied group, supporting the assumption that histological findings might be reasonably extrapolated across the entire study population.

These findings collectively indicate that PASC-vac symptoms in this patient cohort were severe, prolonged, and consistently associated with objective evidence of SFN in a significant proportion of biopsied cases. Furthermore, the overall clinical and diagnostic criteria utilized in this study are consistent with SFN, also in patients without direct skin biopsy confirmation.

### Anti-SARS-CoV-2 receptor antibodies are detected in a limited subset of individuals with post-vaccine PASC

Following reports of potentially serious cardiovascular adverse events post-SARS-CoV-2 vaccination, albeit at a significantly lower incidence than observed with wild-type infection (15, 17, 30, 35) the hypothesis was proposed that these reactions might be associated with the generation of anti-idiotype antibodies (28). To investigate this, we analyzed sera from our patient cohort and a cohort of vaccinated apparently healthy donors (HD) to detect antibodies specific for ACE-2 and Neuropilin-1 (NRP-1), the two known human receptors for SARS-CoV-2 (18–20, 36). ACE-2 reactivity was assessed using a commercial ELISA, while NRP-1 reactivity was evaluated via immunofluorescence and confirmed by confocal microscopy (**Supplementary Figure 1**).

ELISA analysis revealed anti-ACE-2 antibodies in 9 of 71 (12%) patient sera and 4 of 19 (21%) HD sera (p = NS). NRP-1 reactivity was observed in 14 of 71 (20%) patient sera and 1 of 19 (5%) HD sera (p = NS, Fisher’s test) (**Figure 1**). **Supplementary Figure 1** provides confocal microscopy evidence confirming NRP-1 specificity for serum antibodies from PASC-vac patients.

**Figure 3:**
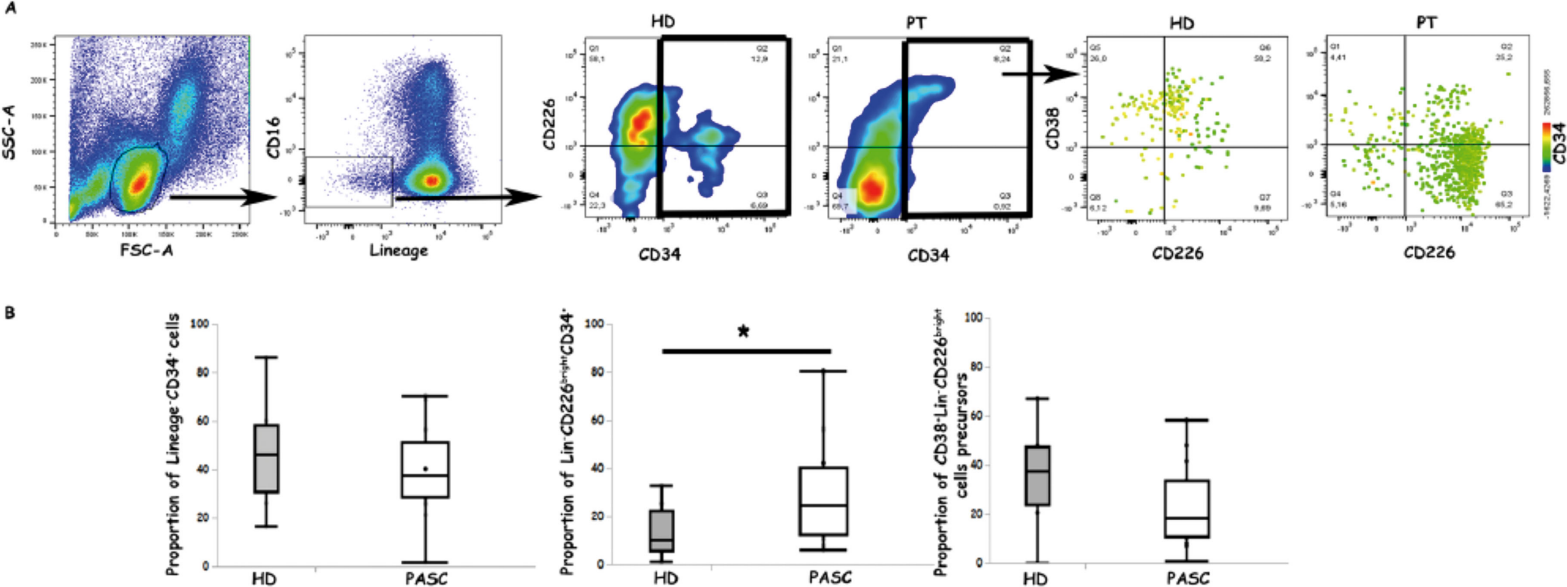
Flow cytometric analysis of inflammatory precursors in PBMC of PASC-vac patients. **(A)** gating strategy and representative dot-plot analysis for the identification of inflammatory CD34^+^ precursors in PBMC (CD34^+^DNAM-1^bright^) in a representative patient and a HD. **(B)** analysis of all CD34^+^, of CD34^+^DNAM-1^bright^ and of CD34^+^CD38^+^DNAM-1^bright^ cells circulating in PBMC of HD and of PASC-vac patients. *p<0.05.

Analysis of the co-occurrence of these autoreactive antibodies in PASC-vac patients demonstrated that anti-NRP-1 serum antibodies were not associated with the presence of anti-ACE-2 antibodies.

Collectively, these findings indicate that anti-NRP-1 antibodies are present in a numerically, though not statistically significantly, higher proportion of PASC-vac patients compared to healthy vaccinated individuals. Furthermore, anti-ACE-2 antibodies were also detected in a subset of vaccinated healthy donors.

### Increased frequency of innate-like T-cells and of inflammatory CD34+ precursors in PASC-vac patients

To investigate potential peripheral blood mononuclear cell (PBMC) subset skewing in post-vaccine PASC (PASC-vac), we performed flow cytometry analysis on PBMC samples from 20 consecutive PASC-vac patients, comparing the results to those obtained from vaccinated healthy donors (HD).

Given prior reports of increased circulating inflammatory CD34^+^ precursors in systemic inflammatory conditions such as HIV, HCV infection, chronic obstructive pulmonary disease (COPD), and non-small cell lung cancer (NSCLC) following chemotherapy/immunotherapy (32, 37, 38), we initially assessed the frequency of these precursors in PASC-vac patients. Flow cytometric analysis revealed comparable total CD34^+^ cell frequencies between the two groups (**Figure 3A**). However, we observed a significant increase in CD34^+^DNAM^bright^ precursors in PASC-vac patients compared to HD (**Figure 3B**). Furthermore, CD38 expression on CD34^+^DNAM-^bright^ cells circulating in HD PBMC was twofold higher than in PASC-vac patients, suggesting a shorter bone marrow residence time and accelerated egress in the latter group (**Figure 3B**).

**Figure 4:**
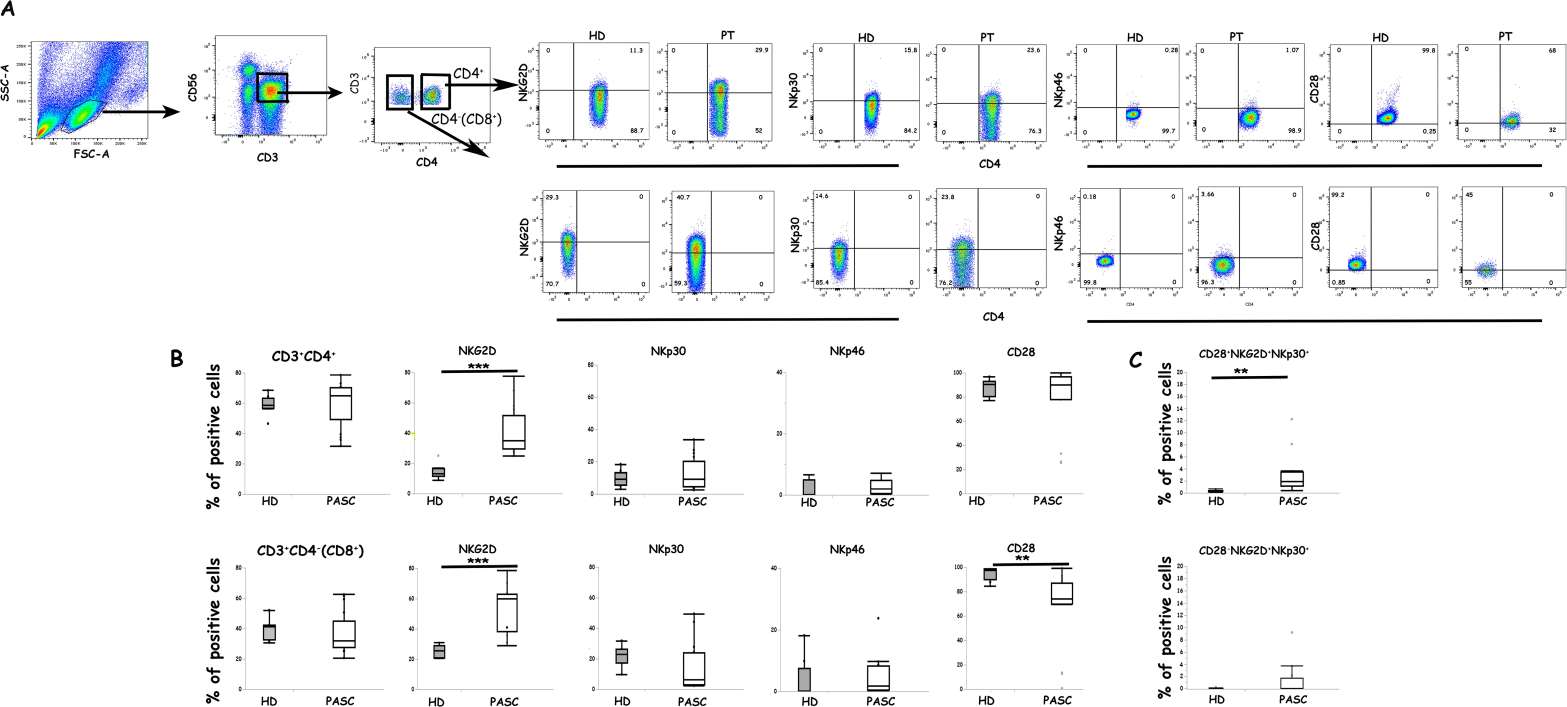
Flow cytometric analysis of CD4^+^ and CD8^+^ T cells in PBMC of PASC-vac patients and of HD. **(A)** gating strategy and representative dot-plot analysis for a patient and a HD. **(B)** box-plot analysis of the expression of NKG2D, NKp30, NKp46 and CD28 on PBMC from PASC-vac patients and HD. **p<0.01; ***p<0.001.

Analysis of CD4^+^ and CD8^+^ (CD4^-^CD3^+^)T-cell populations (**Figures 4A**, gating strategy) demonstrated comparable overall frequencies between PASC-vac and HD groups. Notably, PASC-vac patients exhibited a decreased frequency of CD28^+^CD8^+^ T cells, accompanied by an increased frequency of NKG2D^+^CD8^+^ T cells (**Figure 4B**). When examining CD4^+^ T-cells, we found comparable frequencies of CD28 and activating NK cell receptor expression. However, there was an increased frequency of CD4^+^ T cells simultaneously expressing NKG2D, NKp30, and NKp46. Circulating NK cells did not reveal significant skewing in PASC-vac patients.

Collectively, these data suggest that PASC-vac patients exhibit a state of chronic inflammation. This state is characterized by several key features: increased bone marrow egress of CD34^+^ inflammatory precursors, heightened circulation of T cells expressing activating NK cell receptors, with an increased potential for antigen-independent tissue reactivity.

### Purified inflammatory CD34^+^ precursors isolated from PASC-vac patients generate functional, maturing innate-like T cell progenies *in vitro*

To further investigate the capacity of the increased circulating inflammatory CD34^+^ precursors in PASC-vac patients to generate T and NK cell precursors, we performed an *in vitro* assay. This assay utilized highly purified (>99%) CD34^+^DNAM^bright^ precursors, which were seeded in stem cell culture medium.

Progenitor-derived progenies were identified by optical growth at 14 days post-seeding and subsequently characterized by flow cytometry. This analysis revealed a predominant *in vitro* expansion of T-cell progenies (90.13 ± 5.38%, mean ± SD) and a minority of NK cell progenies (6.8 ± 3.36%, mean ± SD). NK cell progenies exhibited a mature phenotype, with a significant proportion of CD56dim cells (53.63 ± 6.67%, mean ± SD). However, due to their limited frequency, further phenotypic and functional characterization of NK cell progenies was precluded.

**Figure 5:**
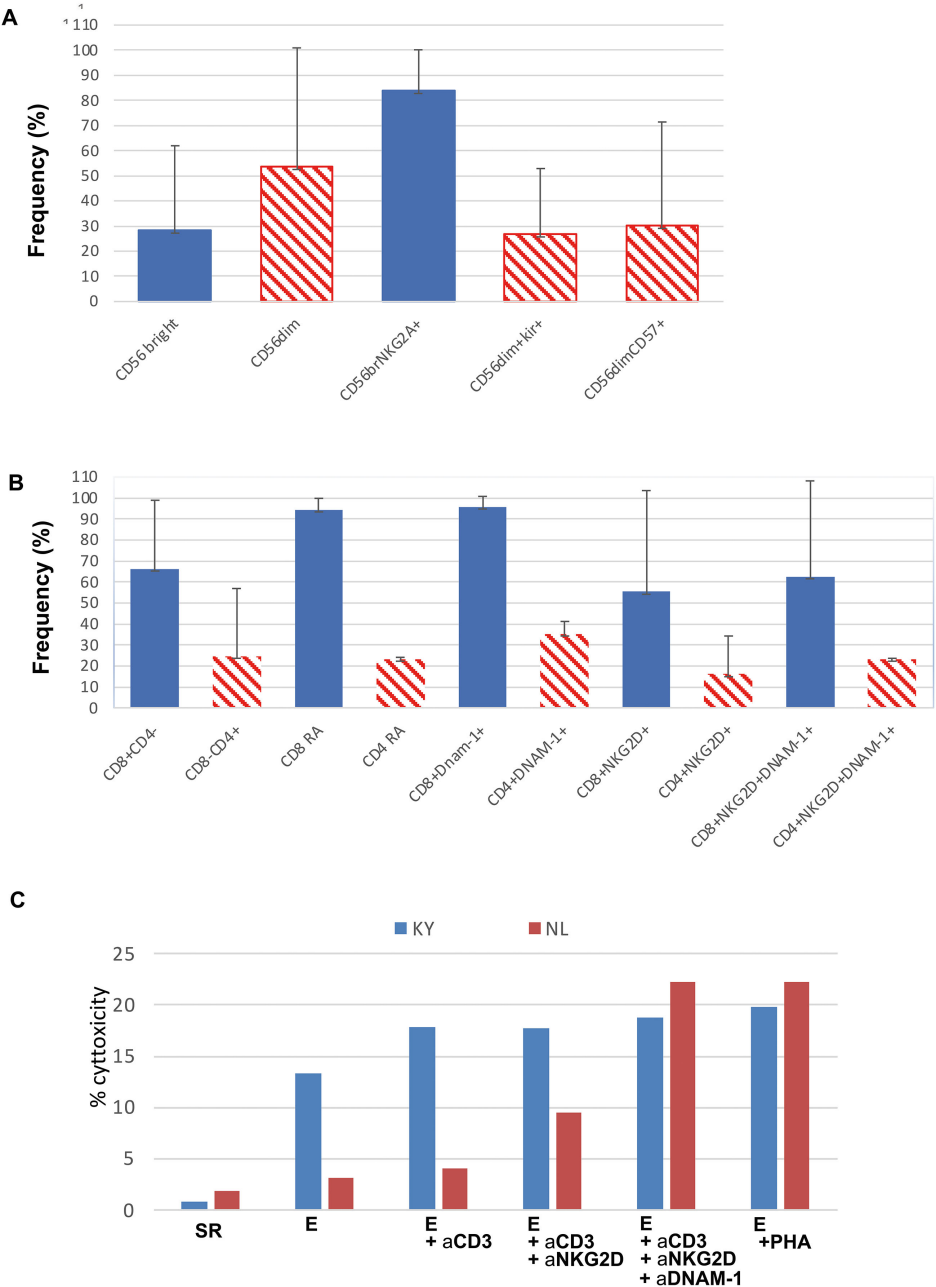
Characterization of *in vitro* grown progenies from highly purified CD34^+^DNAM-1^bright^ cells *in vitro*. **(A)** Characterization of maturation in NK cell progenies **(B)** Expression on T-cell progenies of activating NK cell receptors. **(C)** Cytotoxicity of T-cell progenies expressing activating NK cell receptors in a reverse ADCC assay using indicated mAbs and FcgR+ P815 cells.

T-cell progenies were further analyzed by flow cytometry, demonstrating a predominant CD4^+^ phenotype and a minority of CD3^+^CD8^+^ cells (78.3 ± 11.6% and 21.6 ± 11.6%, mean ± SD, respectively) (**Figure 5A**). T-cell progenies derived from limiting dilution cultures of highly purified CD34^+^DNAM^bright^ precursors displayed expression of the activating NK cell receptors DNAM-1 and NKG2D, while lacking expression of other activating receptors including NKp30, NKp46, and CD16 (**Figure 5B**). Notably, the phenotype of these *in vitro*-generated precursors was consistent with the increased frequencies observed in peripheral PBMC analyses.

Given the potential for CD3^+^ and activating NK cell receptor co-expression to redirect T-cell functional activity towards stressed self-recognition, independently of canonical TCR triggering and antigen presentation, we performed cytotoxicity assays to evaluate this possibility utilizing inflammatory CD34^+^ cell-derived progeny. As shown in **Figure 5C**, CD3^+^CD8^+^ progeny expressing NKG2D and DNAM-1 exhibited enhanced cytotoxic activity in a reverse antibody-dependent cellular cytotoxicity (ADCC) assay in the presence of anti-NKG2D, anti-DNAM-1, and anti-CD3 monoclonal antibodies. Conversely, CD3^+^CD8^+^ progeny lacking these activating receptors did not demonstrate enhanced cytotoxicity.

These findings show that the progenies from inflammatory CD34^+^ precursors that circulate with increased frequency, are enriched in innate-T cells with phenotypes indistinguishable from mature PBMC, and Ag-independent cytotoxic potential.

## Discussion

Here, we report on a cohort of previously healthy patients who experienced the onset of PASC syndrome with Small Fiber Neuropathy (SFN) following SARS-CoV-2 vaccination. PASC-vac/SFN symptoms included both dysautonomic and somatic involvement, frequently distributing to the trunk, face, and head, in addition to typical distal paresthesia. Underlying immune characteristics of PASC-vac included increased circulation of innate-like T cells and elevated inflammatory precursor frequencies, which were found to generate potentially antigen-independent functional innate-like T cells *in vitro*.

Small fiber neuropathy (SFN) is a condition characterized by damage to the small nerve fibers in the peripheral nervous system, specifically the thinly myelinated Aδ fibers and unmyelinated C fibers. These fibers are responsible for transmitting pain, temperature, and autonomic functions. In the present cohort, skin biopsy confirmed SFN diagnosis in patients presenting with compatible symptoms (39).

Our cohort demonstrates a nuanced relationship with ‘classical’ small fiber neuropathy (SFN). On one hand, patients who did not undergo skin biopsy exhibited clinical manifestations—including age at presentation, gender distribution, and qualitative symptom profiles (e.g., burning pain and paroxysmal paresthesias)—that were indistinguishable from those with biopsy confirmation. These features align with historical European SFN cohorts (40), affirming the diagnostic validity of our clinical criteria.

Regarding the clinical symptoms in PASC-vac SFN, the high symptom intensity scores following their abrupt onset post-vaccination, coupled with symptom persistence at elevated levels for over a year, align with previous reports of patients with autoimmune or metabolic SFN in European series (40, 41). This consistency underscores that effective management of pain and dysautonomic symptoms represents a major therapeutic challenge in these patients.

In addition to these overlapping aspects, we identified two distinctive phenotypic deviations in our PASC-vac cohort compared to pre-pandemic SFN presentations (40, 41). First, we observed a predominantly abrupt onset, with symptoms emerging abruptly rather than through the ‘smoldering’ progression over months or years typically reported in the literature (39–41). This rapid onset may suggest an immediate immune-mediated or inflammatory insult to the small fibers rather than a slow neurodegenerative process. Second, our cohort showed a high prevalence of non-length-dependent symptoms, with 63% of patients reporting involvement of the trunk, face, or head—a distribution more frequent than the approximately 20-30% reported in classical SFN series (39, 42). By recognizing these specific temporal and topographical differences within a clinically recognized SFN framework, we can better characterize the unique neurological signature of PASC-vac.

This abrupt symptom onset in otherwise healthy vaccinees raises the possibility that the immune response following antigen introduction, and potentially prolonged immune stimulation (27, 29–32) may be involved in patients who lack other described causes of SFN (40, 41). It should be noted, however, that in the absence of systematic skin biopsy and molecular or biochemical characterization across the entire cohort, it is not possible to definitively establish whether the observed presentation represents the same pathophysiological phenotype as classical SFN. Our data therefore support the presence of a clinical SFN phenotype with substantial overlap, while leaving open the possibility of distinct triggering mechanisms or disease dynamics, including lymphocyte infiltration with nerve destruction and/or functional impairment particularly in those cases without decreased intraepithelial small fiber density. Indeed, a decreased small fiber density does not represent the only requirement for the definition of SFN and, so far, explanations on how a fraction of patients with SFN have normal skin biopsies are lacking. In this respect functional defects may underlie clinical SFN characteristics, in addition to decreased small fiber density. Further, there are few clues as to whether infiltrating lymphocytes are always present around areas with decreased small fiber densities or whether their presence may interfere with peripheral small fiber function even in the absence of decreased fiber densities. While studying lymphocyte infiltrates in skin biopsy was not within our original scope, this emerges as an essential area of inquiry that is a priority for future research to further elucidate the local immune environment in PASC-vac and in general SFN.

While anti-idiotype antibodies reacting with SARS-CoV-2 receptors have been hypothesized as a potential trigger for cardiovascular adverse events in PASC-vac (28) and have been observed at low frequencies in vaccinated (2%) and convalescent (5%) patient sera (43, 44), we could not confirm this in our PASC-vac cohort. The frequency of anti-ACE2 idiotypes in PASC-vac patients was not different from vaccinated healthy donors. Similarly, a non-significant increase in anti-NPR-1 antibodies in PASC-vac patients (20% vs. 5%) suggests this may be only a marginal immune mechanism induced by SARS-CoV-2 vaccination, without a direct relationship to SFN onset in these vaccinees.

On the other hand, a mechanistic link between our immunological findings and the clinical presentation of SFN is provided here by the demonstration of mobilization of pro-inflammatory precursors from the bone marrow with accelerated bone marrow exit, and T-cell perturbations related to autoimmunity.

The co-expression of CD3 and another activating NK cell receptor on T cells is considered a possible mechanism by which antigen-independent, persistent inflammation is maintained in some immune-mediated diseases. For instance, CD4+NKG2D+ T cells in the lamina propria of Crohn’s disease patients are activated by MIC-A expressing cells, contributing to cytotoxicity and mucosal damage (45, 46). CD4+NKG2D+ cells circulate in SLE (47, 48), have been found to be involved in the pathogenesis of SLE by reducing Treg numbers and function (49) and are observed in PBMC and synovial fluid of patients with Rheumatoid Arthritis (50). Thus, our finding in PASC-vac patients of increased circulating frequencies of CD4+NKG2D+DNAM−1+ and CD8+NKG2D+ T cells, and their *in vitro* generation from purified circulating inflammatory CD34+DNAM−1bright precursors, indicates ongoing inflammation with accelerated bone marrow exit of these precursors as shown by reduced CD38 expression alongside an expansion of cells associated with immune-dependent tissue damage.

In NSCLC patients, increased inflammatory CD34+DNAM-1bright precursors circulate after chemotherapy, enter the lungs and generate innate-like T-cell progenies that produce Th1, Th2 and Th17 cytokines including IL17A (38) that has been linked to liver fibrosis induced by NKG2D^+^CD4^+^T cells (51). Similarly, the expansion of innate-like T cells in PASC-vac PBMCs and the increased egress of inflammatory precursors from their bone marrow niche, which *in vitro* generate innate-like T cells, may contribute to small fiber damage through direct cytotoxicity or induction of fibrosis via cytokine expression, thereby leading to SFN.

The present study is limited by the inability to evaluate biopsy specimens for the presence or type of lymphocyte infiltrates in cutaneous biopsies. In a pre-SARS-CoV-2 vaccine era study of skin biopsies from SFN patients, inflammatory cells were present in most SFN samples, with higher gene expression for tumor necrosis factor-α, IL-1β, IL-6, and IL-8 observed in affected skin compared to non-affected skin (52). Additionally, patient selection involved proactive inclusion based on symptom referral, and the study did not include analysis of immunogenetic traits. These factors result in a lack of information on the frequency of observed Vaccine-Associated PASC with SFN and on possible involved immunogenetic traits. This underscores the need for future studies to address these frequencies and mechanisms to improve future vaccine tailoring.

In conclusion, we describe a cohort of patients with post-vaccine PASC satisfying SFN definition criteria, for whom an underlying persistent inflammation (evidenced by CD34+DNAM-1bright cells) was observed, along with a peripheral expansion of innate-like T cells previously linked to other immune-mediated diseases. Although persistent immune stimulation in some vaccine recipients, similar to Long-COVID, may underlie the generation of this skewed immune response and ultimately SFN, the acute onset of disease in some patients underscores the need for a clearer definition of the initiating mechanisms. Further insights into this issue will improve early recognition and patient treatment, the tailoring and targeting of successful mRNA vaccines and limit collateral end-organ damage.

## Data Availability

The raw data supporting the conclusions of this article will be made available by the authors, without undue reservation.
