# Supplementary material for: Immune correlates underlying small fiber neuropathy presenting as vaccine-associated post-acute SARS- coronavirus syndrome
Source: Front Immunol. 2026 Mar 19;17:1752120. doi: 10.3389/fimmu.2026.1752120 (PMC13043346; doi:10.3389/fimmu.2026.1752120)
Supplement: Supplementary file 1 [file DataSheet1.pdf]

Suppl. Figure 1

Confocal microscopy identifying NRP-1 binding of serum Ab of PASC-vac patients.

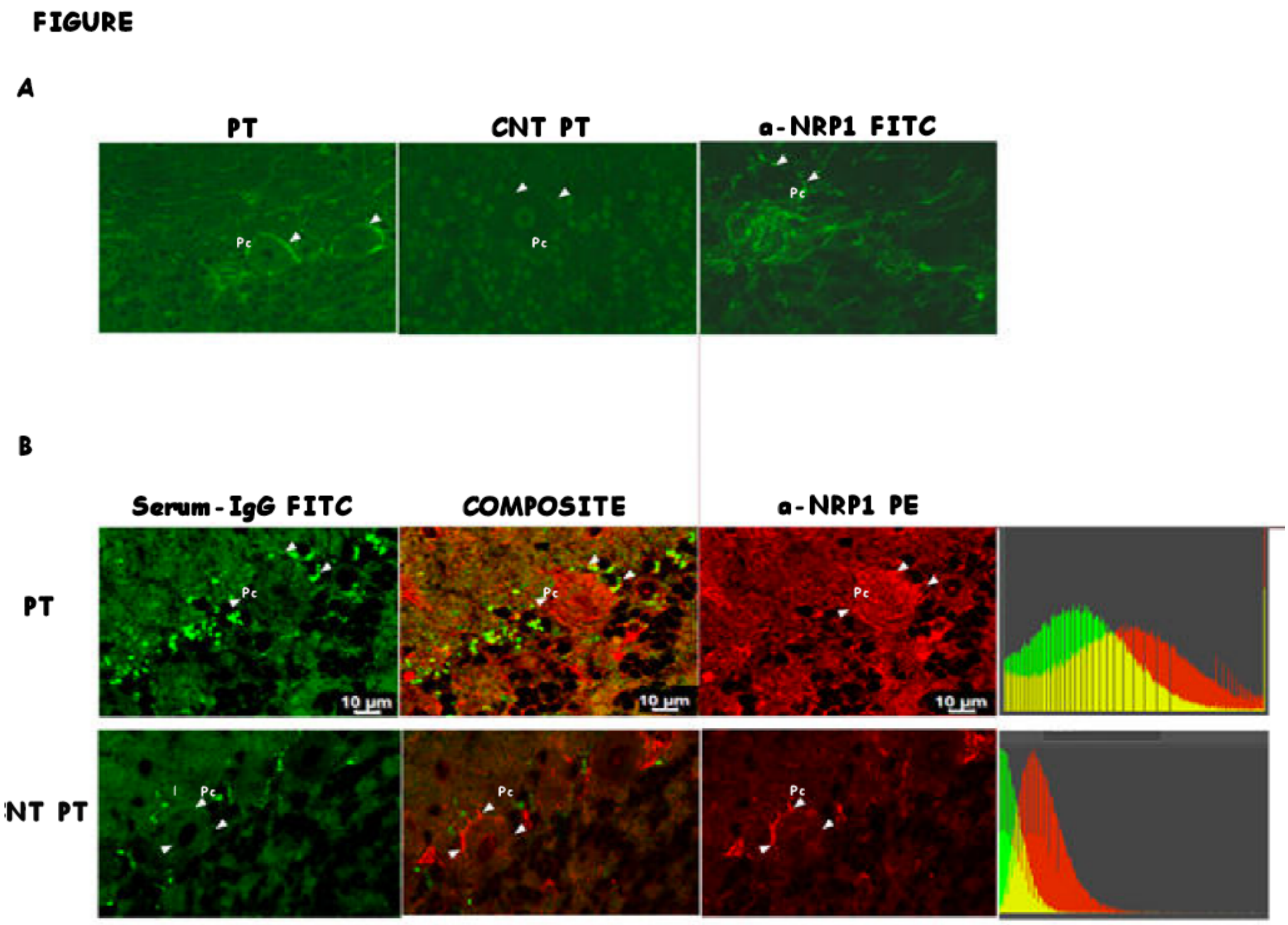

Suppl.Figure 1: Study of the presence of serum anti-NRP1 antibodies in PASC patients and in control healthy donors.

Figure…A.Panel A: IFI analysis on primate cerebellum of serum of PASC patients compared to (Panel B) control healthy patients, secondary total IgG FITC conjugated were used; serum dilution 1:10. Panel C: anti-NRP1 PE-conjugated monoclonal antibodies positive control is shown.

Figure…B: Confocal microscopy analysis of antianti-NRP1 antibodies presence. Green is shown FITC stained serum of PASC patients total IgG antibodies while in red is shown PE.-conjugated monoclonal anti-NRP1 antibodies positive control. Composite panel shown overlay among FITC and PE. Serum dilution 1:10. Images were acquired by Stellaris confocal microscopy (Leica Microsystems). Histograms show degree of overlay of fluorochrome expression for mAb anti-NRP-1 and patient serum. $R^{2=}0,53$; overlap coefficient 0,944 p<0,01 (ImageJ; Fiji).
