# Supplementary material for: Immune correlates underlying small fiber neuropathy presenting as vaccine-associated post-acute SARS- coronavirus syndrome
Source: Front Immunol. 2026 Mar 19;17:1752120. doi: 10.3389/fimmu.2026.1752120 (PMC13043346; doi:10.3389/fimmu.2026.1752120)
Supplement: Supplementary file 2 [file DataSheet2.pdf]

Supplemental Information:

Supplemental Table 1

Clinical characteristics of 71 (patients with biopsy-proven SFN and comparison to patients with PASC (Post-Vaccination) without access to skin biopsy).

Symptoms	Total Number(%)	Those who have undergone biopsy examination (20) Number (%)	Those who have not undergone a biopsy examination. (51) Number (%)	P value
**(Dis-) Autonomic**	61(87.14%)	20(100%)	41(80.39%)	ns
**Orthostatic**	25(35.21%)	7(35%)	18(35.29%)	ns
**Vasomotor**	23(32.39%)	5(25%)	16(31.37%)	ns
**Brain fog**	29(40.84%)	12(42%)	17(33.33%)	ns
**Fatigue**	22(30.98%)	7(35%)	15(29.41%)	ns
**Tinnitus**	20(28.16%)	10(50%)	10(19.60%)	ns
**Dyspnea**	14(19.71%)	5(25%)	9(17.64%)	ns
**Gastrointestinal**	14(19.71%)	5(25%)	9(17.64%)	ns
**Nausea**	6(8.45%)	3(15%)	3(5.88%)	ns
**Diarrhea**	6(8.45%)	2(10%)	4(7.84%)	ns
**Irregular bowel**	6(8.45%)	3(15%)	3(5.88%)	ns
**Constipation**	4 (7.4%)	2(10%)	2(3.92%)	ns
**Skin manifestation**	12(16.9%)	2(10%)	10(19.60%)	ns
**Urinary symptoms**	2(2.81%)	0(0%)	2(3.92%)	-----

Suppl. Table 2:
Clinical characteristics of 71 patients (with biopsy-proven SFN and comparison to patients with PASC (Post-Vaccination) without access to skin biopsy).

Symptoms	Total Number (%)	YES(20) Number (%)	NO(51) Number (%)	P value
**Lower Limb Paresthesia**	56(78.87%)	20(100%)	36(70.58%)	ns
**Upper Limb Paresthesia**	41(57.74%)	15(75%)	26(50.98%)	ns
**Face Paresthesia**	31(43.66%)	8(40%)	23(45.09%)	ns
**Slowing down thought processes**	28(39.43%)	11(55%)	17(33.33%)	ns
**Alteration in tactile sensitivity**	32(54.07%)	11(55%)	21(41.17%)	ns
**Toracic paresthesia**	18(25.35%)	8(40%)	10(19.60%)	ns
