# Supplementary material for: Immune correlates underlying small fiber neuropathy presenting as vaccine-associated post-acute SARS- coronavirus syndrome
Source: Front Immunol. 2026 Mar 19;17:1752120. doi: 10.3389/fimmu.2026.1752120 (PMC13043346; doi:10.3389/fimmu.2026.1752120)
Supplement: Supplementary file 3 [file Table1.docx]

**SUPPLEMENTARY EXPERIMENTAL PROCEDURE**

| **REAGENT or RESOURCE** | **SOURCE** | **IDENTIFIER** |
| --- | --- | --- |
| **Antibodies** | | |
| CD56 PeCy7 | Immunotech-Coulter, Marseille, France | Item N° A21692 |
| CD34 PeCy7 | BD Pharmigen, San Jose, CA, USA | CAT 560710 |
| CD34 FITC | BD Pharmigen, San Jose, CA, USA | CAT 555821 |
| CD38 PerCp/Cy5.5 | BioLegend, San Diego, CA, USA | CAT 303522 |
| CD3 FITC | San Diego, CA, USA | CAT 300306 |
| CD3 BV510 | BD Pharmigen, San Jose, CA, USA | CAT 563109 |
| CD14 BV510 | BD Pharmigen, San Jose, CA, USA | CAT561391 |
| CD19 BV510 | BD Pharmigen, San Jose, CA, USA | CAT 562947 |
| Lineage Cocktail 2 (lin2) (CD3, CD14, CD19, CD20, CD56) FITC | BD Pharmigen, San Jose, CA, USA | CAT 643397 |
| CD16 APC-CY7 | BD Pharmigen, San Jose, CA, USA | CAT 557758 |
| NKG2D PerCp/Cy5.5 | BioLegend, San Diego, CA | CAT 320818 |
| NKp30 Alexa Fuor® 647 | BD Pharmigen, San Jose, CA, USA | CAT 558408 |
| NKP46 HorizonV450 | BD Pharmigen, San Jose, CA, USA | CAT 562099 |
| CD4 PC7 | BD Pharmigen, San Jose, CA, USA | CAT 557852 |
| CXCR4 APC | BD Pharmigen, San Jose, CA, USA | CAT 555972 |
| CD28 BV650 | BD Pharmigen, San Jose, CA, USA | CAT 740593 |
| **Software and Algorithms** | | |
| FlowJo 8.8.7 | FlowJo LLC | www.flowjo.com |
| FlowJo 10.4.0 | FlowJo LLC | www.flowjo.com |
| JMP 10.0.0 | SAS Institute Inc. |  |
| FCSExpress 7 | DeNovo |  |
